# A Digital Program for Daily Life Management With Endometriosis: Pilot Cohort Study on Symptoms and Quality of Life Among Participants

**DOI:** 10.2196/58262

**Published:** 2025-02-28

**Authors:** Zélia Breton, Emilie Stern, Mathilde Pinault, Delphine Lhuillery, Erick Petit, Pierre Panel, Maïa Alexaline

**Affiliations:** 1 Lyv Healthcare Nantes France; 2 Université Paris-Saclay, Gustave Roussy, Inserm, 94805 Villejuif France; 3 GHU Paris Psychiatry & Neurosciences Paris France; 4 Laboratoire de Psychopathologie et Processus de Santé Université Paris Cité Boulogne-Billancourt France; 5 Paris Saint-Joseph Hospital Group EndoSud Ile-de-France Paris France; 6 Clinique Oudinot Paris France; 7 Department of Radiology Fondation Hôpital St Joseph Paris France; 8 Centre de l'Endométriose RESENDO (Réseau Ville-Hopital Endometriose) Paris France; 9 Department of Gynecology-Obstetrics Centre Hospitalier de Versailles Le Chesnay-Rocquencourt France

**Keywords:** digital program, endometriosis, integrative therapies, quality of life, nonpharmacological intervention, daily life, adenomyosis, lesions, women, digital, France, case control, digital health, pilot study, control group, global symptom, burden, depression, neuropathic pain, chronic, multidisciplinary, web based, mobile health, mHealth, intervention

## Abstract

**Background:**

After experiencing symptoms for an average of 7 years before diagnosis, patients with endometriosis are usually left with more questions than answers about managing their symptoms in the absence of a cure. To help women with endometriosis after their diagnosis, we developed a digital program combining user research, evidence-based medicine, and clinical expertise. Structured around cognitive behavioral therapy and the quality of life metrics from the Endometriosis Health Profile score, the program was designed to guide participants for 3 months.

**Objective:**

This cohort study was designed to measure the impact of a digital health program on the symptoms and quality of life levels of women with endometriosis.

**Methods:**

In total, 63% (92/146) of the participants were included in the pilot study, recruited either free of charge through employer health insurance or via individual direct access. A control group of 404 women with endometriosis who did not follow the program, recruited through social media and mailing campaigns, was sampled (n=149, 36.9%) according to initial pain levels to ensure a similar pain profile to participants. Questionnaires assessing quality of life and symptom levels were emailed to both groups at baseline and 3 months. Descriptive statistics and statistical tests were used to analyze intragroup and intergroup differences, with Cohen *d* measuring effect sizes for significant results.

**Results:**

Over 3 months, participants showed substantial improvements in global symptom burden, general pain level, anxiety, depression, dysmenorrhea, dysuria, chronic fatigue, neuropathic pain, and endo belly. These improvements were significantly different from the control group for global symptom burden (participants: mean –0.7, SD 1.6; controls: mean –0.3, SD 1.3; *P*=.048; small effect size), anxiety (participants: mean –1.1, SD 2.8; controls: mean 0.2, SD 2.5; *P*<.001; medium effect size), depression (participants: mean –0.9, SD 2.5; controls: mean 0.0, SD 3.1; *P*=.04; small effect size), neuropathic pain (participants: mean –1.0, SD 2.7; controls: mean –0.1, SD 2.6; *P*=.004; small effect size), and endo belly (participants: mean –0.9, SD 2.5; controls: mean –0.3, SD 2.4; *P*=.03; small effect size). Participants’ quality of life improved between baseline and 3 months and significantly differed from that of the control group for the core part of the Endometriosis Health Profile-5 (participants: mean –5.9, SD 21.0; controls: mean 1.0, SD 14.8; *P*=.03; small effect size) and the EQ-5D (participants: mean 0.1, SD 0.1; controls: mean –0.0, SD 0.1; *P*=.001; medium effect size). Perceived knowledge of endometriosis was significantly greater at 3 months among participants compared to the control group (*P*<.001).

**Conclusions:**

This study’s results suggest that a digital health program providing medical and scientific information about endometriosis and multidisciplinary self-management tools may be useful to reduce global symptom burden, anxiety, depression, neuropathic pain, and endo belly while improving knowledge on endometriosis and quality of life among participants.

## Introduction

### Background

Endometriosis is a chronic, inflammatory disease affecting an estimated 1 in 10 women of childbearing age. It is defined by the presence of endometrial-like tissue outside the uterine cavity [1]. Manifestations of the disease are mainly painful (eg, dysmenorrhea, dyspareunia, dyschezia, and dysuria), with symptoms substantially impacting the quality of life (QOL) of those affected [2]. There is no definitive cure, and available solutions aim to reduce symptoms. The initial solutions are generally hormonal, which are not always well tolerated by patients [1], consistent with their fertility goals, or effective in relieving pain [3]. While surgery was once widely recommended to patients with endometriosis, given the frequent disease recurrence [4], it is now increasingly restricted to selected patients for whom it can be relevant in European countries [5]. Thus, living with endometriosis means learning to deal with the symptoms, using tools other than or in complement to hormonal therapy. After experiencing symptoms for an average of 7 years before diagnosis [6], the lack of an adapted care pathway for symptom management is a crucial unmet need of the patients [7]. Various nonpharmacological interventions have been studied to help with symptom management, such as dietary changes, physical activity, sex therapy, and mind-body interventions, which seem pertinent for the daily management of endometriosis [8-12]. Digital tools may offer a solution to enhance accessibility to and observance of nonpharmacological interventions toward endometriosis symptom reduction.

### Prior Work

Developing a program to improve QOL requires the construction of content corresponding to what can modulate the QOL of women with endometriosis. Numerous studies to assess changes in patients’ QOL have been using the Endometriosis Health Profile (EHP) score as a reference for almost 20 years [13]. The EHP is a standardized tool for measuring QOL. Initially developed with 30 items (EHP-30) [14], a shortened version with 5 items (ie, EHP-5) was also validated [15]. The following EHP components were taken into account in developing the programs: pain, control and powerlessness, emotional well-being, social support, self-image, work life, infertility and children management, sexual relationships, and relation to the medical profession.

Cognitive behavioral therapy (CBT) is a well-supported treatment for various disorders [16]. CBT’s goal is to help individuals better understand the relationship and interaction between their emotions, cognitions (received ideas), and behavior to be able to manage their symptoms and quit the vicious cycle that contributing factors may create through behavior change (develop coping mechanisms), cognitive restructuring (restructure maladaptive thoughts), and emotion regulation (relaxation and acceptance) [17-19]. It was found to improve the QOL for patients with chronic pain conditions [20-23], especially by encouraging a problem-solving attitude in the patients receiving the treatment [24]. Internet-based CBT programs have been proven effective for a variety of disorders [25-29]. When considering CBT-based programs for chronic pain [18,30], the following components are found: psychoeducation on pain mechanisms and biopsychosocial model, goal settings, CBT-based behavior skills for managing pain (eg, pleasant activities and sleep hygiene), emotion regulation and mind-body interventions (eg, relaxation methods and mindfulness), cognitive restructuring, and long-term action plan for maintenance and anticipation of obstacles.

### Goal of This Study

A digital solution for endometriosis symptom management was developed using a CBT approach based on EHP items. The digital support solution focused on the scientific and medical state of the art around 5 nonpharmacological interventions (disease education including pain mechanism, diet, adapted physical activity, well-being and mental health, and sexual health). The *School of Endo* digital program was developed and made available in France to help women with endometriosis after their diagnosis.

This cohort study was designed to measure the impact of a digital health program on the symptoms and QOL levels of women with endometriosis. The study also aimed to provide insights into the value of a digital program for the day-to-day management of endometriosis and to address the lack of real-life data studies for digital support in endometriosis.

## Methods

### Development of a Digital Health Program

To develop a digital health program that adequately addresses the needs of patients with endometriosis, several steps were followed, where product development and scientific research complemented each other [31]. As the first step, 120 semistructured interviews and >100 web-based questionnaires with patients and health professionals were conducted to understand the needs of women with endometriosis. According to this user research, the primary need expressed by women with endometriosis was to find help with the daily management of symptoms, especially pain. A small exploratory preliminary program was developed and tested by women to complement the user research. A scientific rationale and advice from medical experts completed the user research. User research, evidence-based medicine, and clinical expertise were combined to enable the development of the program [32].

The structure of the program’s content was based on CBT and the QOL items of the EHP score, and participants were advised to follow it for 3 months. The program was accessible from November 2022 for women with endometriosis aged >18 years, either at no fee through employer health insurance (*Mutuelle Générale de l’Education Nationale*) or individual direct payment. Program participants were invited to join the pilot study on a voluntary basis.

This program sought to provide evidence-based information and tools to empower patients on symptom management through a wide range of content: videos, exercises, written content, live sessions, quizzes, and a community-based platform. A total of 12 endometriosis experts were involved in the construction of the program, constituting a multidisciplinary team.

Scientific rationale highlighted some factors of interest for the program. In an Australian study on 484 women with endometriosis, dietary changes were reported among the most effective strategies in terms of reducing self-reported pain associated with the disease [8]. Optimizing nutrition is a nonpharmacological intervention that can be used to manage the symptoms of endometriosis. The positive effects of physical activity have been documented for various health conditions, including mental health (improved cognitive function, enhanced QOL, improved sleep, and reduced signs of anxiety and depression) and chronic pain [12,33,34]. According to current research, psychological interventions and mind-body approaches are promising for improving QOL and alleviating pain, anxiety, depression, stress, and fatigue in women with endometriosis [9,10]. The impact of endometriosis on sex life harms patients’ self-esteem. Sex therapy and psychological interventions can be very beneficial during treatment to improve QOL [11].

The program addressed every aspect of the disease through 5 sections: disease education including pain mechanism, diet, adapted physical activity, well-being and mental health, and sexual health. It started with a section on knowledge of the disease, with an introduction to the program and the multidisciplinary approach. Psychoeducation was focused on defining the disorder, its causes, and its impacts. It helped the participant generate a model of their disorder and pain to obtain a representation of a functional analysis of the factors perpetuating the symptoms. The first part of the program concluded with educating the participants on setting goals and initiating change and provided tools to help them set their goals.

CBT content is included throughout the program. Each section mentioned subsequently contains education, advice, and tools created by health care professionals in the field.

The diet section covered step-by-step behavior change through good mealtime habits, from breakfast to dinner, with the help of calendars to fill in. This section provided information on why and how to make dietary changes with endometriosis, particularly on the role of diet in inflammation and digestive symptoms. A section dedicated to identifying inflammatory foods was also available to participants.

The sections dedicated to physical activity presented different ways to stay active while experiencing symptoms and suggested various methods, including yoga, Pilates, physiotherapy exercises, and daily habits, to move the body. A 30-minute yoga session and a Pilates session were available each week. These sessions focused mainly on pelvic mobility. Physiotherapy complemented the physical activity with ventral breathing exercises and a focus on the perineum and abdominal muscles.

The mental health section focused mainly on education and prevention to help with emotional regulation. Relaxation practices, sophrology exercises, Beck columns, and a weekly diary to initiate behavior changes were proposed.

The sexuality and intimacy section featured physiotherapy exercises, sex therapy tools, and advice to rediscover pleasure and self-confidence in sexual relations.

**Figure 1:**
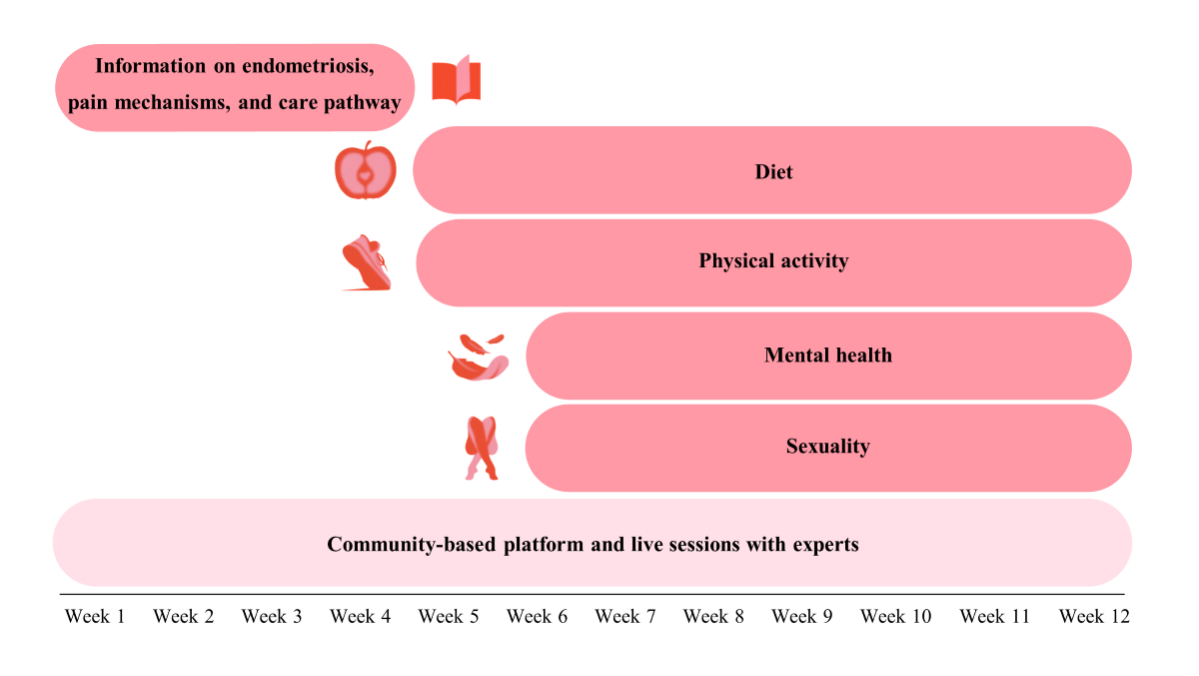
Sections contained in the 3-month digital program offered to participants with endometriosis on which this cohort study is based.

The program allowed participants to create their toolbox and action plan. Section contents were delivered weekly for 3 months and were available for a further 3 months (Figure 1).

### Ethical Considerations

A study protocol was established in compliance with French regulations, reviewed by the Lyv Healthcare company’s ethics committee, and filed with the French public structure for studies on health data: Health Data Hub (N° F20221114165253). Participants answered the questionnaires on a voluntary basis after being informed about the research project objectives and were provided with contact information to exercise their rights. At any time, participants had the possibility to opt out. All collected data were pseudonymized and stored in compliance with health data security standards (Health Data Hosting certification, h*ébergeurs de données de santé*). No personal information was shared with third parties, and all data analysis was performed on deidentified datasets. No compensation was provided to either program participants or the control group for their participation in the study.

### Impact of the Digital Health Program

#### Questionnaires

Our study compared 2 groups, distinct in terms of exposure to the digital program, both having participants with endometriosis. The study is considered a cohort study because the allocation of respondents to the exposure criterion was not controlled for the research participant, and there was no prospective assignment. Two web-based questionnaires were sent to program participants via email links and reminders on the program’s community-based platform. The first questionnaire was sent at the time of inclusion in the program (time point 0 [T0]) and the second one after 3 months (T0+3 mo), with 2 weeks to submit their answers. Participants completed the questionnaires on their own, requiring approximately 15 minutes to complete. Questionnaires were structured into 2 parts, including a main part (53 questions) and a bonus part (24 questions), explaining differences in the sample size for some outcomes. Both questionnaires were electronically tested before diffusion. A back button allowed respondents to review and modify their answers, while IP addresses were used to ensure that each participant was a unique visitor, with only the first submission used for analysis.

#### Population Sample

Women were free to choose whether to take part in the program, with no obligation to complete it in full. Program participants were given the freedom to answer the questionnaires. This pilot study included participants who completed the main part entirely at baseline and 3 months.

Only women who reported being diagnosed with endometriosis could answer the questionnaires. The diagnosis could be clinical, imagery based, or surgical. To record the effect of active use of the program on participants, the threshold of following at least half of the proposed content types was retained. As a result, among the participants, only women who declared having tested >50% of the proposed content type in the program were included.

The terms woman and women are used in this paper. It should be noted that people with a uterus may or may not identify with these terms and experience endometriosis. Sex and gender were not criteria for inclusion in the study.

#### Control Group

A control group of women with endometriosis who did not follow the program was recruited via social media and a health insurance provider email campaign (*Mutuelle Générale de l’Education Nationale*). The women in the control group were volunteers, motivated solely by the desire to contribute to advancing research, as no incentives, prizes, or rewards were offered for their participation. The control group received the same questionnaires as program participants during the same periods to eliminate external effects on measured parameters.

The control group was sampled on the initial pain level to have a similar profile between them and program participants. Women were selected from the control group by stratifying the sample to reproduce the distribution of pain levels among program participants. QOL answers were not selected for this stratification as these questions were part of bonus questionnaires. A random sample of women from the control group was selected so that the same proportion was represented for each modality of the participants’ general pain level. The proportional sample was thus selected, reducing the number of women in the control group to 149.

#### Study Outcomes Measures

##### Numeric Rating Scales

Numeric Rating Scales (NRSs) are 11-point scales, ranging from 0 (no pain or symptom) to 10 (intense pain or symptom), primarily used to measure symptom intensity. NRS evaluated the level of overall pain, anxiety, depression, dysmenorrhea, dyspareunia, dyschezia, dysuria, chronic pelvic pain, gastrointestinal disorders, chronic fatigue, neuropathic pain, and endo belly. Endo belly is a common term used by patients with endometriosis to describe the cyclic bloating of the abdomen [35] as “uncomfortable or painful, often accompanied by a sensation of abdominal fullness,” and which “often forces women with endometriosis to wear loose clothing” [36]. Endo belly affects >80% of women with gastrointestinal symptoms due to endometriosis [37].

Women without menstruation did not respond to the dysmenorrhea NRS, and women without sexual activities did not respond to the dyspareunia NRS, which explains differences in sample size for these outcomes.

An average of each of these NRSs was used to determine a global symptom burden score, ranging from 0 to 10.

##### EHP-5 Scale

EHP-5 is a QOL scale specific to endometriosis. A main score is calculated out of 100 (100=poor QOL and 0=good QOL), based on 5 items with 5 modalities (scored as 0, 25, 50, 75, and 100). The main score is the average of the scores for the 5 items. A modular score is calculated out of 100 (100=worst QOL and 0=best QOL), based on 6 optional 5-modality items (scored as 0, 25, 50, 75, and 100). The score is the average of the scores for the 6 items.

##### EQ-5D Scale

EQ-5D is a QOL scale nonspecific to endometriosis. It comprises 5 items with 5 modalities scored from 1 to 5. The concatenation of the 5 scores gives a combination. Each combination ranging from 11111 to 55555 has a numerical equivalent between –0.53 and 1. EQ-5D score ranges from –0.53 (poor QOL) to 1 (good QOL).

#### Statistical Analysis

Symptoms were considered to have evolved (improved or deteriorated) if scores had changed by >2 points compared with T0 [38]. QOL was considered to have evolved (improved or deteriorated) if the score had changed by >15% compared with T0 [39]. Otherwise, outcomes were considered stable.

The minimum sample size was calculated through a feasibility study power analysis for outcomes between program participants and the control group. This feasibility study was carried out on a preliminary program among 55 participants to identify ways to improve the program and observe symptoms and QOL levels. Global pain on the NRS was assumed by a mean (SD) of 5.9 (2.3). Assuming a difference of at least 2 points, a power (1–β) of 0.80, α=.05, and using a 2-sided 2-sample test, 11 program participants and 11 participants from the control group would be required to detect group differences. QOL was measured on the EHP-5, assumed by a mean (SD) of 53.6 (19.0). Assuming a change in score of at least 15%, a power of 0.80, α=.05, and using a 2-sided 2-sample test, 13 program participants and 13 participants from the control group would be required for detecting between-group differences. On the basis of power analyses, it was planned to include answers from at least 13 program participants and 13 from the control group.

Descriptive statistics were carried out (number of participants, percentage, mean, and SD). Statistical tests used to measure the significance of intragroup evolution were the chi-square test and the Fisher exact test for discrete variables, the Wilcoxon signed rank test for paired and nonparametric continuous variables, and the paired 2-tailed Student *t* test for parametric continuous variables. Statistical tests used to measure between-group differences (participants vs controls) were the chi-square test and the Fisher exact test for discrete variables, the Mann-Whitney *U* test for unpaired and nonparametric continuous variables, and the unpaired 2-tailed Student *t* test for parametric continuous variables. Furthermore, we applied the Benjamini-Hochberg method to adjust for multiple comparisons. This method controls the false discovery rate, offering a balance between minimizing false positives and maintaining statistical power. Cohen *d* tests were performed to measure effect size when the result was statistically significant. If Cohen *d*>|0.2|, the effect size was considered small, medium if Cohen *d*>|0.5|, and large if Cohen *d*>|0.8|. Each outcome was studied separately. A sensitivity analysis was conducted to note the impact of program follow-up according to baseline QOL level. For this, linear regression models and interaction tests were used with the EHP-5 core QOL level (good QOL: 0 to 32, medium QOL: 33 to 65, and low QOL: 66 to 100). Statistical analysis was performed with SAS software (version 9.4; SAS Institute).

## Results

### Population Description

**Figure 2:**
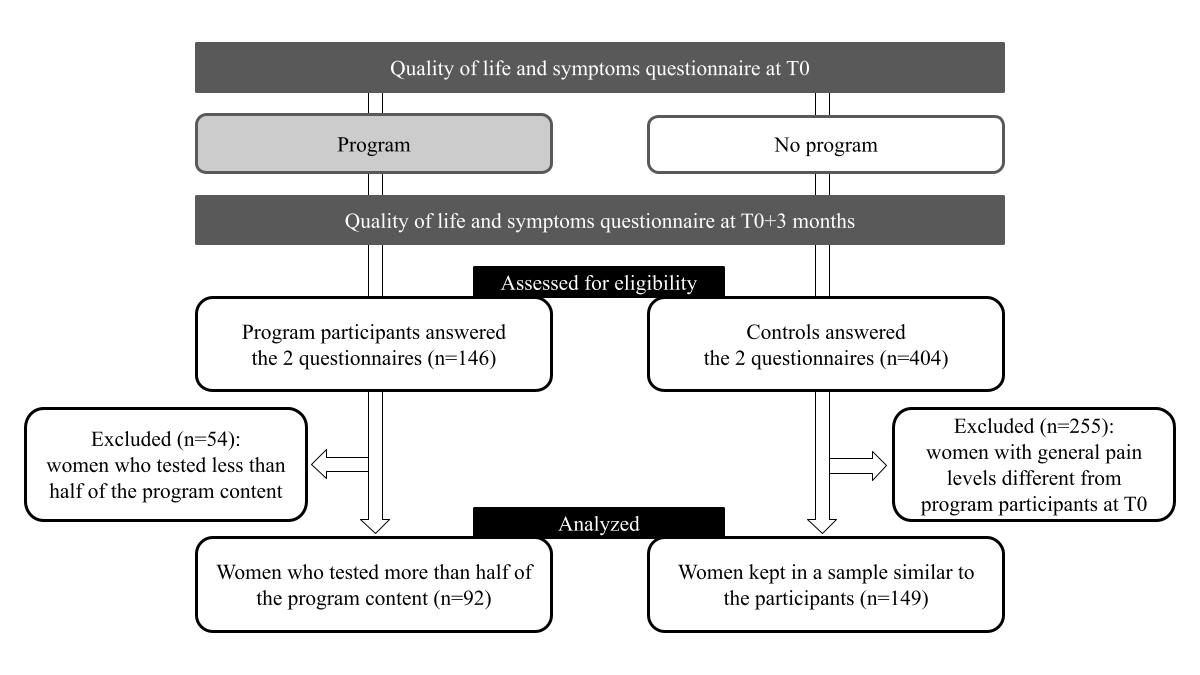
Cohort study flow diagram: cohort study responders were divided according to their participation in the 3-month digital program for women with endometriosis. T0: time point 0.

The pilot study was based on the responses to the main questionnaire part from 92 program participants diagnosed with endometriosis and 149 women diagnosed with endometriosis who did not follow the program, constituting the control group (Figure 2).

Respondents to the questionnaires took part in this study on a voluntary basis. Table 1 presents the characteristics of the program participants and those of the control group at baseline. On average, participants were aged 36.7 (SD 6.8) years and were diagnosed with the disease when they were aged 32.5 (SD 6.7) years. In total, 26% (24/92) of the participants were experiencing or had experienced infertility problems. There were no substantial differences in socioeconomic characteristics or pain levels at the time of inclusion compared to the control group. Participants had a slightly poorer QOL at the time of inclusion than those in the control group (*P*=.04) and were more often on a continuous hormonal pill (*P*=.046). The baseline symptom levels and QOL of program participants are provided in Multimedia Appendix 1.

Analyses were based on the answers given by participants to the initial questionnaire and the one at 3 months.

**Table 1:** Sociodemographic, clinical, pain-related, and quality of life baseline characteristics of program participants and those in the control group with endometriosis (N=241).

			Program participants (n=92)		Control group (n=149)		*P* value
**Sociodemographic characteristics**							
	Age (y), mean (SD)	36.7 (6.8)		36.6 (7.2)		.91	
	Single status, n (%)	23 (25)		29 (19.5)		.64	
	Does not feel surrounded and feels very alone, n (%)	6 (6.5)		11 (7.4)		.92	
	Perceived financial situation (0=very insecure to 10=very comfortable), mean (SD)	5.4 (1.7)		5.4 (1.9)		.73	
	Number of children, mean (SD)	0.7 (1.0)		0.8 (1.0)		.34	
	Number of pregnancies, mean (SD)	1.1 (1.4)		1.3 (1.5)		.11	
**Diagnosis of endometriosis and treatment**							
	Experience of the pathway to diagnosis (0=very poor to 10=very good), mean (SD)	2.7 (2.4)		3.4 (2.7)		.09	
	Age at diagnosis (y), mean (SD)	32.5 (6.7)		31.6 (6.8)		.21	
	Age at first symptoms (y), mean (SD)	18.7 (7.3)		19.9 (8.7)		.56	
	Time between first set of symptoms and diagnosis, mean (SD)	13.5 (8.9)		11.5 (8.6)		.10	
	Hormonal treatment, n (%)	53 (57.6)		65 (43.6)		.046	
**Symptoms and quality of life**							
	Experienced infertility problems, n (%)	24 (26.1)		47 (31.5)		.37	
	Dysmenorrhea level (0=no pain to 10=worst pain), mean (SD)	7.3 (1.8)		6.5 (2.3)		.12	
	Global symptom burden (0=no symptom to 10=worst symptom intensity), mean (SD)	5.3 (1.6)		5.0 (1.6)		.17	
	Proportion of global symptom burden ≥8, n (%)	5 (5.4)		4 (2.7)		.31	
	Quality of life: EHP-5^a^ (0=best to 100=worst), mean (SD)	53.8 (20.4)^b^		45.2 (20.3)^c^		.04	
	Proportion of quality of life: EHP-5 ≥80, n (%)	11 (12)		9 (6)		.15	

^a^EHP-5: Endometriosis Health Profile-5.

^b^n=39 program participants.

^c^n=65 individuals from the control group.

### Impact on Endometriosis Symptoms

Initially, the participants’ most severe symptom was chronic fatigue (mean 7.5, SD 2.1), followed by dysmenorrhea (mean 7.3, SD 1.8) and anxiety (mean 6.5, SD 2.2; Table 2).

Overall, there was a tendency for all symptoms to improve during the 3 months of following the program (Table 3). For some symptoms, the evolution was not significant compared with the control group: dyspareunia, dyschezia, chronic pelvic pain, gastrointestinal disorders, overall pain, dysuria, and chronic fatigue. Over 3 months, the global symptom burden, general level of pain, anxiety, depression, dysmenorrhea, dysuria, chronic fatigue, neuropathic pain, and endo belly improved significantly for the program participants. These improvements were significantly different compared to the control group for global symptom burden (*P*=.048; small effect size), anxiety (*P*<.001; medium effect size), depression (*P*=.04; small effect size), neuropathic pain (*P*=.004; small effect size), and endo belly (*P*=.03; small effect size).

**Table 2:** Outcome evaluation at baseline and 3 months for program participants versus those in the control group with endometriosis.

Health outcome	Program participants (n=92), n (%)	Control group (n=149), n (%)	T0^a^					T0+3 mo			
			Program participants, mean (SD)	Control group, mean (SD)	Participants vs control group		Program participants, mean (SD)		Control group, mean (SD)	Participants vs control group	
					*P* value	Cohen *d*				*P* value	Cohen *d*
Global symptom burden	90 (97.8)	147 (98.7)	5.3 (1.6)	5.0 (1.6)	.17	0.1^b^	4.6 (1.6)		4.7 (1.8)	.75	–0.0^b^
Overall pain	90 (97.8)	147 (98.7)	6.2 (2.5)	6.0 (2.4)	.68	0.1^b^	5.5 (2.6)		5.3 (2.6)	.66	0.1^b^
Anxiety	90 (97.8)	147 (98.7)	6.5 (2.2)	5.9 (2.7)	.12	0.2^c^	5.5 (2.9)		6.1 (2.7)	.12	–0.2^c^
Depression	89 (96.7)	147 (98.7)	4.0 (3.0)	4.2 (3.2)	.87	–0.0^b^	3.3 (3.1)		4.2 (3.3)	.05	–0.3^c^
Dysmenorrhea	35 (38)	74 (49.7)	7.3 (1.8)	6.5 (2.3)	.12	0.3^c^	5.6 (2.6)		5.8 (2.6)	.54	–0.1^b^
Dyspareunia	43 (46.7)	84 (56.4)	3.9 (2.4)	4.1 (2.8)	.90	–0.0^b^	3.1 (2.5)		3.5 (2.7)	.47	–0.2^c^
Dyschezia	90 (97.8)	147 (98.7)	3.7 (3.2)	4.2 (2.8)	.19	–0.2^c^	3.3 (2.7)		3.6 (2.9)	.60	–0.1^b^
Dysuria	90 (97.8)	147 (98.7)	2.2 (2.9)	1.8 (2.5)	.36	0.2^c^	1.6 (2.0)		1.6 (2.4)	.35	0.0^b^
Chronic pelvic pain	90 (97.8)	147 (98.7)	5.2 (2.8)	4.8 (2.7)	.22	0.2^b^	4.8 (2.8)		4.5 (2.7)	.48	0.1^b^
Gastrointestinal disorders	90 (97.8)	147 (98.7)	5.9 (2.7)	5.3 (2.7)	.10	0.2^c^	5.7 (2.3)		5.1 (2.8)	.13	0.2^c^
Chronic fatigue	90 (97.8)	147 (98.7)	7.5 (2.1)	7.0 (2.2)	.09	0.2^c^	6.8 (2.3)		6.8 (2.3)	.77	–0.0^b^
Neuropathic pain	90 (97.8)	147 (98.7)	5.4 (3.1)	4.6 (3.2)	.09	0.2^c^	4.3 (2.9)		4.5 (3.0)	.47	–0.1^b^
Endo belly	90 (97.8)	147 (98.7)	6.2 (2.5)	5.7 (2.7)	.09	0.2^c^	5.4 (2.7)		5.3 (2.7)	.92	0.0^b^
QOL^d^: EHP-5^e^ core part	39 (42.4)	65 (43.6)	53.8 (20.4)	45.2 (20.3)	.04	0.0^b^	47.9 (19.4)		46.2 (21.5)	.88	0.0^b^
QOL: EHP-5, modular part	39 (42.4)	64 (43)	34.9 (13.0)	28.7 (18.4)	.05	0.4^c^	26.7 (15.4)		26.4 (18.8)	.59	0.0^b^
QOL: EQ-5D score	33 (35.9)	80 (53.7)	0.8 (0.1)	0.8 (0.2)	.06	–0.2^c^	0.9 (0.1)		0.8 (0.2)	.44	0.0^b^

^a^T0: time point 0.

^b^No effect size.

^c^Small effect size.

^d^QOL: quality of life.

^e^EHP-5: Endometriosis Health Profile-5.

**Table 3:** Continuous outcomes evolution between baseline and 3 months for program participants versus the control group with endometriosis.

Health outcome	Program participants, mean (SD)	Within program participants		Control group, mean (SD)	Within control group		Program participants vs control group		Benjamini-Hochberg threshold
		*P* value	Cohen *d*^a^		*P* value	Cohen *d*	*P* value	Cohen *d*	
Global symptom burden	–0.7 (1.6)	*<.001* ^b^	*0.4* ^c^	–0.3 (1.3)	*.004*	*0.2* ^c^	*.048*	–*0.3*^c^	0.022
Overall pain	–0.7 (2.9)	*.02*	*0.3* ^c^	–0.7 (2.5)	*<.001*	*0.3* ^c^	.84	—^d^	0.050
Anxiety	–1.1 (2.8)	*<.001*	*0.4* ^c^	0.2 (2.5)	.51	—	*<.001*	–*0.5*^e^	*0.003*
Depression	–0.9 (2.5)	*.002*	*0.3* ^c^	0.0 (3.1)	.92	—	*.04*	–*0.3*^c^	0.019
Dysmenorrhea	–1.9 (2.8)	*<.001*	*0.7* ^e^	–0.7 (2.4)	*.01*	*0.3* ^c^	.05	—	0.025
Dyspareunia	–0.5 (2.8)	.28	—	–0.7 (2.2)	*.007*	*0.3* ^c^	.69	—	0.044
Dyschezia	–0.4 (2.3)	.16	—	–0.6 (2.7)	*.004*	*0.2* ^c^	.47	—	0.038
Dysuria	–0.5 (2.3)	*.045*	*0.2* ^c^	–0.1 (2.3)	.41	—	.22	—	0.034
Chronic pelvic pain	–0.5 (3.0)	.15	—	–0.3 (2.6)	.29	—	.53	—	0.041
Gastrointestinal disorders	–0.2 (2.7)	.36	—	–0.2 (2.3)	.31	—	.76	—	0.047
Chronic fatigue	–0.7 (2.5)	*.02*	*0.3* ^c^	–0.2 (2.3)	.29	—	.14	—	0.031
Neuropathic pain	–1.0 (2.7)	*<.001*	*0.4* ^c^	–0.1 (2.6)	.58	—	*.004*	–*0.4*^c^	*0.009*
Endo belly	–0.9 (2.5)	*.002*	*0.3* ^c^	–0.3 (2.4)	.21	—	*.03*	–*0.2*^c^	0.013
QOL^f^: EHP-5^g^ core part	–5.9 (21.0)	.09	—	(14.8)	.59	—	*.03*	–*0.4*^c^	0.016
QOL: EHP-5, modular part	–8.2 (16.9)	*.004*	–*0.5*^e^	–2.5 (11.1)	.06	—	.10	—	0.028
QOL: EQ-5D score	0.1 (0.1)	*.01*	*0.5* ^e^	–0.0 (0.1)	.08	—	*.001*	*0.7* ^e^	*0.006*

^a^The effect size was considered small if Cohen *d* >|0.2|, medium if Cohen *d* >|0.5|, and large if Cohen *d* >|0.8|.

^b^Values in italics indicate significant *P* values.

^c^Small effect size.

^d^Not applicable.

^e^Medium effect size.

^f^QOL: quality of life.

^g^EHP-5: Endometriosis Health Profile-5.

When looking at symptom evolution (improvement, stability, or deterioration), the distribution was not significantly different between the 2 groups for dyspareunia, dyschezia, dysuria, chronic pelvic pain, gastrointestinal disorders, and chronic fatigue (Table 4). Global symptom burden improved in 20% (18/90) of the program participants versus 6.1% (9/147) in the control group (*P*=.003). Anxiety levels improved in 42% (38/90) and deteriorated in 11% (10/90) of the program participants versus 26.5% and 28.6%, respectively, in the control group (*P*=.002). Depression deteriorated less in program participants (9/89, 10%) than in the control group (41/147, 27.9%; *P*=.003). Neuropathic pain improved more in program participants (37/90, 41%) than in the control group (34/147, 23.1%; *P*=.02), as did endo belly (37/90, 41% vs 36/147, 24.5%; *P*=.03).

Actively following the digital program for 3 months was associated with a significant improvement in global symptom burden, anxiety, depression, neuropathic pain, and endo belly perception among program participants when compared to the control group.

**Table 4:** Evolution of symptoms between baseline and 3 months for program participants versus the control group with endometriosis.

Health outcome		Program participants				Control group				Program participants vs control group, *P* value			Benjamini-Hochberg threshold	
		Improvement	Stable	Deterioration	Improvement		Stable	Deterioration						
**Global symptom burden (n=90 participants; n=147 controls)**											*.003* ^a^			*0.009*
	Participants, n (%)	18 (20)	68 (75.6)	4 (4.4)	9 (6.1)		133 (90.5)	5 (3.4)						
	Points, mean (SD)	–3.1 (0.9)	–0.3 (1.0)	2.6 (0.7)	–2.9 (0.9)		–0.2 (1.0)	2.5 (0.5)						
**Overall pain (n=90 participants; n=147 controls)**											*.09*			0.029
	Participants, n (%)	34 (37.8)	39 (43.3)	17 (18.9)	43 (29.3)		85 (57.8)	19 (12.9)						
	Points, mean (SD)	–3.8 (1.7)	0.1 (0.8)	3.3 (1.3)	–3.7 (1.6)		–0.1 (0.7)	3.2 (1.5)						
**Anxiety (n=90 participants; n=147 controls)**											*.002*			*0.006*
	Participants, n (%)	38 (42.2)	42 (46.7)	10 (11.1)	39 (26.5)		66 (44.9)	42 (28.6)						
	Points, mean (SD)	–3.6 (1.8)	–0.1 (0.7)	4.2 (1.9)	–2.9 (1.2)		0.2 (0.7)	3.0 (1.4)						
**Depression (n=89 participants; n=147 controls)**											*.003*			*0.012*
	Participants, n (%)	28 (31.5)	52 (58.4)	9 (10.1)	42 (28.6)		64 (43.5)	41 (27.9)						
	Points, mean (SD)	–3.7 (1.7)	0.0 (0.8)	3.0 (1.5)	–3.5 (1.6)		0.0 (0.7)	3.7 (2.1)						
**Dysmenorrhea (n=35 participants; n=74 controls)**											.07			0.024
	Participants, n (%)	19 (54.3)	14 (40)	2 (5.7)	24 (32.4)		39 (52.7)	11 (14.9)						
	Points, mean (SD)	–4.0 (2.1)	0.1 (0.7)	3.0 (0.0)	–3.3 (1.6)		–0.2 (0.8)	2.9 (1.1)						
**Dyspareunia (n=43 participants; n=84 controls)**											.49			0.038
	Participants, n (%)	13 (30.2)	21 (48.8)	9 (20.9)	25 (29.8)		48 (57.1)	11 (13.1)						
	Points, mean (SD)	–3.9 (1.6)	0.0 (0.8)	3.1 (1.5)	–3.4 (1.6)		0.0 (0.7)	2.3 (0.6)						
**Dyschezia (n=90 participants; n=147 controls)**											.63			0.047
	Participants, n (%)	23 (25.6)	51 (56.7)	16 (17.8)	46 (31.3)		75 (51)	26 (17.7)						
	Points, mean (SD)	–3.3 (1.5)	–0.1 (0.7)	3.1 (1.1)	–3.5 (1.7)		–0.1 (0.8)	3.3 (2.1)						
**Dysuria (n=90 participants; n=147 controls)**											.38			0.035
	Participants, n (%)	24 (26.7)	55 (61.1)	11 (12.2)	28 (19.1)		97 (66)	22 (15)						
	Points, mean (SD)	–3.3 (1.7)	–0.1 (0.5)	3.5 (1.4)	–3.4 (1.5)		0.0 (0.5)	3.6 (1.6)						
**Chronic pelvic pain (n=90 participants; n=147 controls)**											.30			0.032
	Participants, n (%)	33 (36.7)	33 (36.7)	24 (26.7)	44 (29.9)		69 (46.9)	34 (23.1)						
	Points, mean (SD)	–3.7 (1.6)	0.0 (0.7)	3.3 (1.2)	–3.2 (1.6)		0.0 (0.8)	3.1 (1.2)						
**Gastrointestinal disorders (n=90 participants; n=147 controls)**											.90			0.050
	Participants, n (%)	27 (30)	42 (46.7)	21 (23.3)	42 (28.6)		73 (49.7)	32 (21.8)						
	Points, mean (SD)	–3.2 (1.3)	–0.1 (0.8)	3.4 (1.6)	–2.8 (1.1)		0.0 (0.8)	3.0 (1.3)						
**Chronic fatigue (n=90 participants; n=147 controls)**											.49			0.041
	Participants, n (%)	29 (32.2)	46 (51.1)	15 (16.7)	37 (25.2)		81 (55.1)	29 (19.7)						
	Points, mean (SD)	–3.4 (1.6)	–0.1 (0.8)	2.9 (1.4)	–3.1 (1.4)		0.0 (0.7)	2.9 (1.5)						
**Neuropathic pain (n=90 participants; n=147 controls)**											*.02*			0.015
	Participants, n (%)	37 (41.1)	39 (43.3)	14 (15.6)	34 (23.1)		84 (57.1)	29 (19.7)						
	Points, mean (SD)	–3.5 (1.6)	–0.2 (0.7)	3.1 (1.7)	–3.4 (1.6)		0.0 (0.7)	3.6 (1.8)						
**Endo belly (n=90 participants; n=147 controls)**											*.03*			0.021
	Participants, n (%)	37 (41.1)	37 (41.1)	16 (17.8)	36 (24.5)		81 (55.1)	30 (20.4)						
	Points, mean (SD)	–3.4 (1.4)	–0.1 (0.9)	3.3 (1.8)	–3.5 (1.8)		0.0 (0.7)	2.7 (1.1)						

^a^Italicization indicates significant *P* values.

### Impact on QOL

With all 3 scores used (EHP-5 core part, EHP-5 modular part, and EQ-5D), active program participants showed an improvement in their QOL after 3 months on the program (Table 2). This evolution observed between 0 and 3 months significantly differed from the control group for the core part of the EHP-5 (*P*=.03; small effect size) and the EQ-5D (*P*=.001; medium effect size; Table 3). Therefore, the use of the program was associated with an improvement of QOL in participants with endometriosis.

When looking at the types of evolution (improvement, stability, or deterioration) in QOL, an improvement of the EQ-5D and the EHP-5 core part was observed in 2 and 3 times more women in the program participants than in the control group, respectively (Table 5). However, the difference in the types of evolution was not significant between the 2 groups for EQ-5D (*P*=.58) and the core part of EHP-5 (*P*=.07). A significant difference in the types of evolution between the 2 groups was observed for the modular part of the EHP-5 (*P*=.02), with an improvement in 31% (12/39) of the program participants versus 11% (7/64) of the controls.

**Table 5:** Evolution of quality of life (QOL) score items and perceived knowledge between baseline and 3 months for program participants versus the control group with endometriosis.

Health outcome		Program participants				Control group				Program participants vs control group, *P* value			Benjamini-Hochberg threshold	
		Improvement	Stable	Deterioration	Improvement		Stable	Deterioration						
**QOL: EHP-5^a^ core part (n=39 participants; n=65 controls)**											.07			0.026
	Participants, n (%)	16 (41)	17 (43.6)	6 (15.4)	13 (20)		40 (61.5)	12 (18.5)						
	Points, mean (SD)	–25.0 (8.2)	–0.9 (6.9)	30.8 (11.6)	–18.1 (3.8)		0.5 (6.6)	23.3 (11.3)						
**QOL: EHP-5 modular part (n=39 participants; n=64 controls)**											*.02* ^b^			0.018
	Participants, n (%)	12 (30.8)	24 (61.5)	3 (7.7)	7 (10.9)		54 (84.4)	3 (4.7)						
	Points, mean (SD)	–28.1 (9.3)	–2.4 (6.1)	25.0 (8.3)	–23.2 (3.3)		–1.3 (6.9)	23.6 (2.4)						
**QOL: EQ-5D score (n=33 participants; n=80 controls)**											.58			0.044
	Participants, n (%)	2 (6.1)	31 (93.9)	0 (0)	2 (2.5)		78 (97.5)	0 (0)						
	Points, mean (SD)	0.4 (0.1)	0.0 (0.1)	—^c^	0.4 (0.1)		–0.0 (0.1)	—						
Perceived knowledge (n=90 participants; n=148 controls), n (%)		47 (52.2)	41 (45.6)	2 (2.2)	21 (14.2)		119 (80.4)	8 (5.4)	*<.001*			*0.003*		

^a^EHP-5: Endometriosis Health Profile-5.

^b^Values in italics indicate significant *P* values.

^c^Not applicable.

To better understand these evolutions in QOL, we focused on the items that make up the QOL scores (Table 6). There was a significant difference at baseline (*P*=.03; small effect size) in the core part of the EHP-5 score on the item concerning control and powerlessness. Program participants were more affected than those in the control group. This significant difference disappeared at 3 months. Besides, after 3 months, there was a significant improvement in the sexual relationship item (*P*=.02; small effect size) in the modular part of the EHP-5 score, which was not observed in the control group. Participants were less often worried about pain during sexual intercourse after 3 months on the program than before starting the program. Finally, the usual activity item in the EQ-5D score improved nearly significantly for the program participants (*P*=.05; small effect size) after 3 months, which was not the case in the control group, with participants feeling less limited in their usual activities after 3 months on the program.

The benefits of a digital program also depend on how it is used; in this study, it was found that the greater the frequency of use or the greater the diversity of sections consulted, the greater the improvement in symptoms or QOL (Multimedia Appendices 2 and 3). The results did not show any statistically significant correlations between the viewing of action-oriented content and the program’s effectiveness (Multimedia Appendix 3).

**Table 6:** Quality of life (QOL) score items evaluation at baseline and 3 months for program participants versus the control group with endometriosis.

Health outcome		Program participants									Control group								Program participants vs control group						
		T0^a^, mean (SD)		T0 +3 mo, mean (SD)		*P* value		Cohen *d*		T0, mean (SD)			T0 +3 mo, mean (SD)		*P* value		Cohen *d*	T0, *P* value			T0, Cohen *d*		T0+3 mo, *P* value		T0+3 mo, Cohen *d*
**QOL: EHP-5^b^ core part**																									
	Pain	1.4 (1.1)	1.2 (1.0)		.17		—^c^		1.2 (1.0)			1.2 (1.0)		.95		—		.25		—		.99		—	
	Control and powerlessness	2.5 (1.4)	2.1 (1.2)		.06		—		1.9 (1.2)			1.9 (1.2)		.93		—		.03		0.4^d^		.62		—	
	Emotional well-being	2.5 (1.0)	2.3 (0.9)		.39		—		2.3 (1.0)			2.3 (1.1)		.59		—		.79		—		.90		—	
	Social support	2.3 (1.0)	2.1 (0.9)		.19		—		1.9 (1.2)			2.0 (1.1)		.64		—		.15		—		.87		—	
	Self-image	2.2 (1.3)	2.0 (1.4)		.46		—		1.8 (1.2)			1.9 (1.4)		.48		—		.13		—		.80		—	
**QOL: EHP-5 modular part**																									
	Work life	1.2 (1.1)	0.9 (1.1)		.37		—		0.9 (1.2)			0.8 (1.3)		.46		—		.13		—		.21		—	
	Taking care of children	2.2 (1.2)	2.1 (1.1)		.19		—		1.4 (1.1)			1.3 (1.2)		.99		—		.03		0.7^e^		.04		0.7^e^	
	Sexual relationships	2.2 (1.5)	1.7 (1.4)		.02		–0.4^d^		1.7 (1.4)			1.7 (1.4)		.16		—		.15		—		.99		—	
	Medical profession	1.3 (1.2)	0.6 (0.8)		.06		—		1.2 (1.3)			1.1 (1.4)		.66		—		.81		—		.21		—	
	Treatment	2.4 (1.0)	2.2 (1.2)		.25		—		2.1 (1.3)			1.9 (1.4)		.24		—		.32		—		.36		—	
	Infertility	2.2 (1.6)	2.5 (1.4)		.66		—		1.9 (1.7)			2.1 (1.7)		.68		—		.45		—		.52		—	
**QOL: EQ-5D score**																									
	Mobility	1.4 (0.7)	1.2 (0.5)		.11		—		1.5 (0.8)			1.5 (0.8)		.62		—		.87		—		.06		—	
	Self-care	1.1 (0.2)	1.0 (0.2)		.99		—		1.1 (0.4)			1.1 (0.4)		.99		—		.61		—		.17		—	
	Usual activities	2.2 (0.9)	1.9 (0.9)		.05		–0.4^d^		1.9 (0.9)			1.9 (0.8)		.63		—		.05		—		.92		—	
	Pain or discomfort	2.9 (0.8)	2.5 (0.7)		.06		—		2.5 (1.0)			2.5 (1.0)		.64		—		.06		—		.47		—	
	Anxiety or depression	2.6 (0.9)	2.4 (1.0)		.19		—		2.4 (0.9)			2.6 (1.0)		.04		0.2^d^		.28		—		.52		—	
	VAS^f^ (0=worst to 100=best QOL)	58.8 (17.1)	63.0 (15.7)		.23		—		63.4 (20.7)			62.8 (19.2)		.79		—		.13		—		.99		—	

^a^T0: time point 0.

^b^EHP-5: Endometriosis Health Profile-5.

^c^Not applicable.

^d^Small size effect.

^e^Medium size effect.

^f^VAS: visual analog scale.

### Impact on Perceived Knowledge of Endometriosis

Initially, there was no significant difference in perceived knowledge between program participants and the control group (*P*=.74; Table 7). In total, 22% (20/92) of the program participants felt that they knew little about the disease, 71% (65/92) had good knowledge, and 8% (7/92) considered themselves experts (vs 34/149, 22.8%; 99/149, 66.4%; and 16/149, 10.7%, respectively for the control group). At 3 months, none of the program participants considered themselves as knowing little about the disease (0/92, 0%); 64% (59/92) considered themselves as having good knowledge, and 36% (33/92) considered themselves experts (vs 25/149, 16.8%; 104/149, 69.8%, and 20/149, 13.4%, respectively, for the control group). The perceived knowledge of endometriosis was significantly different at 3 months between the 2 groups (*P*<.001; Table 5). As expected, the program seemed to improve perceived knowledge of endometriosis.

**Table 7:** Perceived knowledge on endometriosis at baseline and 3 months for program participants versus the control group. The significance for program participants versus control group is P=.74 at T0 and P<.001 at T0+3 months.

Perceived knowledge on endometriosis	Program participants (n=92), n (%)			Control group (n=149), n (%)	
	T0^a^	T0+3 mo	T0		T0+3 mo
I know little	20 (21.7)	0 (0)	34 (22.8)		25 (16.8)
I have good knowledge, but I am not an expert	65 (70.7)	59 (64.1)	99 (66.4)		104 (69.8)
I consider myself an expert	7 (7.6)	33 (35.9)	16 (10.7)		20 (13.4)

^a^T0: time point 0.

### Multiple Comparisons and Sensitivity Analysis

To address the potential for type I errors due to multiple testing in this pilot analysis, we applied the Benjamini-Hochberg method to adjust significance thresholds while maintaining statistical power. This approach controls the false discovery rate and is particularly suited for exploratory analyses.

Table 1 illustrates that the QOL levels, as assessed by the EHP-5 core part, significantly differed between the 2 groups at baseline (*P*=.04). However, the proportion of participants reporting poor QOL was similar in both groups (*P*=.15). To explore the potential influence of these baseline differences on the evolution of outcomes, we conducted interaction analyses using linear regression models, as detailed in Multimedia Appendix 4. These analyses revealed that baseline QOL differences primarily affected the evolution of dyschezia. Specifically, dyschezia showed more significant improvement in program participants compared to the control group, particularly among women with good baseline QOL. In addition, baseline QOL levels influenced changes in the EHP core part score at 3 months. This score improved significantly among program participants with a distinct pattern compared to the control group, especially for women with good QOL at baseline. Conversely, improvements were more pronounced in the control group than in the program participants for women with moderate or poor QOL at baseline.

## Discussion

### Principal Findings

This pilot study of 92 program participants and 149 individuals in the control group shows that following a 3-month digital health program for the self-management of endometriosis symptoms is associated with a significant reduction in endometriosis-related symptoms (anxiety, depression, neuropathic pain, and endo belly perception), a reduction of the global symptom burden, and a significant improvement in the participants’ knowledge of endometriosis. Furthermore, following the program seemed to be associated with an improvement in QOL. Combining CBT approaches and EHP-focused programs in a digital tool has proved useful in inducing symptom relief and a better QOL.

### Comparison With Prior Work

Previous studies have shown that patients often use nonpharmacological interventions to cope with their symptoms [8,40]. However, only one short-term study has examined the value of combining these nonpharmacological interventions as part of a multidisciplinary approach for QOL with endometriosis [41], and a recent publication tested the application over 3 months [42]. Instead, most published studies analyzed the benefits of such interventions individually. These studies, among other things, have underpinned the development of our proposed program, which offers patients various tools and solutions to manage their symptoms.

As confirmed by our results, CBT has been found to be helpful in managing pain and improving the scores of depression and QOL in patients with endometriosis [43-45]. New studies are also beginning to be conducted on the benefits of psychological and relaxation interventions for patients with endometriosis, thus far demonstrating improvement in pain [46], stress [47], anxiety, depression, and QOL [48,49]. The improvement of mental health–related symptoms in our results might be in part due to the psychological and relaxation approaches included in our program. Given the link between mental health and QOL in women with endometriosis [50], future research should explore the use of more comprehensive scales for anxiety and depression. Furthermore, nutrition and physical activity have been studied separately and proved to have an impact on endometriosis symptoms, stress, anxiety, and QOL [51-56]. Finally, although sexual issues are a prevalent symptom, sex education for women with endometriosis has only been assessed in one study, where an improvement in sexual function and quality of sexual life was observed [57]. Interestingly, in our study, the EHP-5 score for the sexual intercourse apprehension item decreased for participants, notwithstanding persisting dyspareunia. This highlights a change in the perception of sexual anxiety after 3 months, despite the persistence of pain. Digital programs for the management of endometriosis should study the long-term effect of sex education content on dyspareunia, both in terms of symptoms and perception.

While the efficacy and relevance of each intervention for the management of endometriosis symptoms have thus been confirmed by previous research, a multidisciplinary approach is in line with current guidelines; therefore, it should be encouraged for women with endometriosis and more generally for those who experience chronic pain [58,59]. Future studies should seek to determine the optimal combination of nonpharmacological interventions according to patient profiles to find the right balance between diet, physical activity, stress management, sleep, and environmental enrichment on top of their medical care [60,61].

In terms of methodology, our study assessed each symptom using numerical scales to simplify user responses and limit attrition rates. For QOL, we used 2 different scales: the EQ-5D, which is nonspecific to endometriosis, and the EHP-5, which is specific to endometriosis. These were previously compared in a French study [39], which concluded that while both scales were appropriate for assessing the QOL of women with endometriosis, the EHP-5 was better at assessing health-related QOL, especially regarding medical treatment and the intensity of dysmenorrhea. According to our results, the EHP-5 does seem to better discriminate with regard to endometriosis, which echoes the conclusions of the French study [39]. However, regardless of the scale used, following our digital program was associated with an improved QOL. This was true for 2 to 3 times more participants than individuals in the control group. Our study used a 15% threshold to define a change in QOL, allowing stringent evolutions to be highlighted. Program participants had a poorer QOL level at baseline compared to the control group (despite a similar pain profile). However, after 3 months of following the program, program participants caught up with the QOL level of the control group.

Digital tools are a way to bring multidisciplinary interventions to patients, enhancing their accessibility. Studies on digital interventions for various conditions have shown positive results, such as improvements in anxiety and depression through CBT-based approaches [62], symptom management and QOL enhancements via mindfulness-based stress-reduction programs [63], or better health outcomes through digital personalized diets [64]. Similarly, multidisciplinary digital programs have demonstrated significant benefits across diverse chronic conditions, including improved QOL and symptom management. For instance, internet-based programs have been shown to enhance knowledge, increase exercise habits, and reduce heart failure symptoms [65], while personalized digital care programs have improved the QOL in adults with autoimmune diseases and post–COVID-19 condition [66]. Digital support programs have also improved self-reported QOL in patients with rheumatoid arthritis [67] and managing symptoms in patients with cancer [68]. In our study, we observed that a digital tool using a CBT approach is effective in helping patients manage a condition, in this case, endometriosis. This may have to do with the fact that digital technology can bring about a different perception and thus reduce the stigma surrounding intimate issues associated with endometriosis, such as psychological or sexuality aspects [28,69-71]. These findings underscore the potential of multidisciplinary digital programs to relieve symptoms, improve QOL, and enhance disease-related knowledge across various chronic conditions.

For digital therapeutics to achieve their purpose, they must meet patient needs in terms of content while optimizing patient adherence and fulfilling prescriber requirements. The content of our program was based on EHP components to address subjects that were relevant to patients with endometriosis and was approved by health care specialists. Other studies have looked at digital technology for managing endometriosis or pelvic pain and highlighted the need for educational content, psychological and social support, and patient empowerment in particular [40]. As for patient adherence, to enhance digital therapeutic efficiency, it is crucial that future studies analyze specific factors influencing patient engagement in a digital program, such as trust, interactions, and consideration [72].

Keeping patients engaged to increase their frequency of use and guiding them through nonpharmacological, multidisciplinary interventions are therefore key to a program’s efficiency. Guidelines and studies encourage the multidisciplinary management of endometriosis. However, only 2 studies on the same intervention, evaluating the QOL after 2 and 12 weeks following a multimodal program, have been conducted in the context of digital intervention [41,42]. Our study contributes to answering the need for new research on the multidisciplinary management of endometriosis and the development of programs to make such management accessible [73,74].

### Limitations and Strengths

Our study is subject to several limitations. It is based on voluntary participation only, with no compulsory questionnaires and no obligation to complete the program. It also includes a selection bias, with no information as to whether the profiles of the women who answered the questionnaires were similar to the overall profiles of the women who took part in the program. Although the participants in this study was not randomized, differences between program participants and the control group were minimized by selecting a control group sample with pain levels similar to those of program participants (despite a different initial QOL level, the effect of which was analyzed in sensitivity analyses). Using the Benjamini-Hochberg method, we were able to control the false discovery rate. Nevertheless, we interpreted results with borderline *P* values cautiously, acknowledging the increased risk of type I errors inherent in multiple testing.

Furthermore, participants self-reported their diagnosis of endometriosis, which may be clinical, imaging based, or surgical. However, one can assume that women who do not have endometriosis would not attend the program and would not spend time filling in research questionnaires.

A strength of the program is that it was designed based on user research, scientific rationale, and medical expertise. Our approach, targeting EHP components and using CBT, allowed the development of an effective digital tool for the self-management of endometriosis symptoms. In addition, the use of 2 different questionnaires on QOL showed that following the program was associated with an improvement of the health-related QOL (EQ-5D) and the QOL specific to endometriosis (EHP-5). Very little amount of data exists on the reliability and efficiency of a digital program for the management of endometriosis symptoms. Our study is the first step in identifying key factors to be considered for developing a digital health program for the daily management of endometriosis and in demonstrating its positive impact on patient symptoms and QOL.

### Conclusions

Our results suggest that a digital health program providing medical and scientific information about endometriosis as well as multidisciplinary self-management tools may be a helpful and effective resource for women to manage life with endometriosis alongside their medical care. Hence, a digital program for endometriosis that combines integrative solutions, focuses on EHP components, and uses a CBT approach can enhance patient care for those with endometriosis.

## Appendix

Multimedia Appendix 1 Distribution of symptom levels and quality of life for endometriosis program participants at baseline.

Multimedia Appendix 2 The evolution of outcomes between baseline and 3 months for all endometriosis program participants versus the control group according to the content thresholds of the program tested.

Multimedia Appendix 3 The evolution of outcomes between baseline and 3 months for all endometriosis program participants according to the sections thresholds of the program tested.

Multimedia Appendix 4 Beta coefficients and 95% CIs of nonadjusted linear regression models evaluating associations between endometriosis program participation and health outcomes and interaction tests according to baseline quality of life levels (Endometriosis Health Profile-5 core part).

## Abbreviations

CBT: cognitive behavioral therapy
EHP: Endometriosis Health Profile
NRS: Numeric Rating Scale
QOL: quality of life
T0: time point 0

## Data Availability

The datasets generated and analyzed during this study are not publicly available due to the consent agreements made with participants, which did not include provisions for public data release but are available from the corresponding author on reasonable request.
