# Supplementary figures and images for: A Digital Program for Daily Life Management With Endometriosis: Pilot Cohort Study on Symptoms and Quality of Life Among Participants
Source: JMIR Form Res. 2025 Feb 28;9:e58262. doi: 10.2196/58262 (PMC11909486; doi:10.2196/58262)

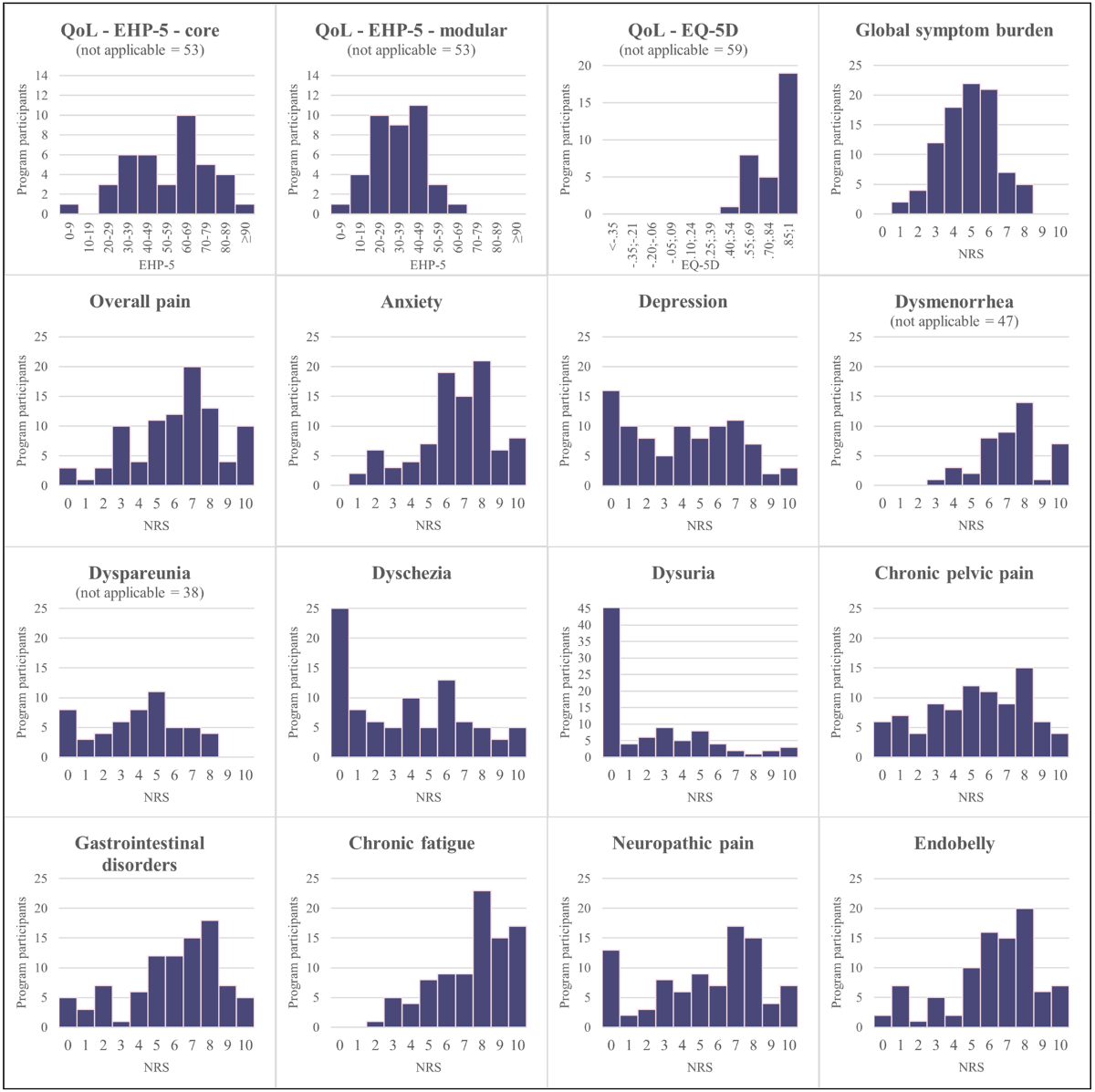

Supplement: Multimedia Appendix 1 [file formative_v9i1e58262_app1.png]
