# Supplementary material for: A Digital Program for Daily Life Management With Endometriosis: Pilot Cohort Study on Symptoms and Quality of Life Among Participants
Source: JMIR Form Res. 2025 Feb 28;9:e58262. doi: 10.2196/58262 (PMC11909486; doi:10.2196/58262)
Supplement: Multimedia Appendix 2 [file formative_v9i1e58262_app2.docx]

Multimedia appendix 2. Evolution of outcomes between baseline and 3 months for all endometriosis program participants vs. control group according to the content thresholds of the program tested.

|  |  | **N** | | **Participants** | | | **Controls** | | | **Participants vs. Controls (a)** | | | **Participants** | | | **Controls** | | | **Participants vs. Controls repartition** |
| --- | --- | --- | --- | --- | --- | --- | --- | --- | --- | --- | --- | --- | --- | --- | --- | --- | --- | --- | --- |
|  | **Proportion of program content tested** | **Participants** | **Controls** | **T0**  **(mean ±SD)** | **T0 + 3 months (mean ±SD)** | **Evolution T0 ; T0 + 3 months (mean ±SD)** | **T0**  **(mean ±SD)** | **T0 + 3 months (mean ±SD)** | **Evolution T0 ; T0 + 3 months (mean ±SD)** | **T0**  **(*P* ; Cohen's d)** | **T0 + 3 months (*P* ; Cohen's d)** | **Evolution T0 ; T0 + 3 months (P ; Cohen's d)** | **Improvement (%)** | **Stable (%)** | **Deterioration (%)** | **Improvement (%)** | **Stable (%)** | **Deterioration (%)** | ***P*** |
|  | 1/4 | 49 | 65 | 53.8 ±20.7 | 48.8 ±20.7 | -5.0 ±19.1 | 45.2 ±20.3 | 46.2 ±21.5 | 1.0 ±14.8 | .18 ; 0.0 N | .92 ; 0.1 N | .045 ; -0.4 S | 35% | 53% | 12% | 20% | 62% | 18% | .08 |
| **QoL - EHP-5 - core** | 1/3 | 41 | 65 | 54.0 ±19.9 | 48.7 ±19.2 | -5.4 ±20.7 | 45.2 ±20.3 | 46.2 ±21.5 | 1.0 ±14.8 | .16 ; 0.0 N | .97 ; 0.1 N | .049 ; -0.4 S | 39% | 46% | 15% | 20% | 62% | 18% | .02 |
|  | 1/2 | 39 | 65 | 53.8 ±20.4 | 47.9 ±19.4 | -5.9 ±21.0 | 45.2 ±20.3 | 46.2 ±21.5 | 1.0 ±14.8 | .04 ; 0.0 N | .88 ; 0.0 N | .03 ; -0.4 S | 41% | 44% | 15% | 20% | 62% | 18% | .02 |
|  | 2/3 | 28 | 65 | 56.3 ±19.7 | 48.0 ±20.1 | -8.2 ±20.5 | 45.2 ±20.3 | 46.2 ±21.5 | 1.0 ±14.8 | .10 ; 0.0 N | .74 ; 0.0 N | .01 ; -0.6 M | 50% | 36% | 14% | 20% | 62% | 18% | .02 |
|  | 1/4 | 115 | 147 | 5.2 ±1.7 | 4.6 ±1.8 | -0.6 ±1.5 | 5.0 ±1.6 | 4.7 ±1.8 | -0.3 ±1.3 | .25 ; 0.1 N | .83 ; -0.0 N | .09 ; - | 17% | 80% | 3% | 6% | 91% | 3% | .03 |
| **Global symptom burden** | 1/3 | 99 | 147 | 5.4 ±1.6 | 4.7 ±1.7 | -0.7 ±1.6 | 5.0 ±1.6 | 4.7 ±1.8 | -0.3 ±1.3 | .71 ; 0.1 N | .88 ; 0.0 N | .06 ; - | 18% | 78% | 4% | 6% | 91% | 3% | .01 |
|  | 1/2 | 90 | 147 | 5.3 ±1.6 | 4.6 ±1.6 | -0.7 ±1.6 | 5.0 ±1.6 | 4.7 ±1.8 | -0.3 ±1.3 | .17 ; 0.1 N | .75 ; -0.0 N | .048 ; -0.3 S | 20% | 76% | 4% | 6% | 91% | 3% | .003 |
|  | 2/3 | 57 | 147 | 5.4 ±1.5 | 4.7 ±1.6 | -0.7 ±1.6 | 5.0 ±1.6 | 4.7 ±1.8 | -0.3 ±1.3 | .16 ; 0.1 N | .99 ; -0.0 N | .10 ; - | 17% | 81% | 2% | 6% | 91% | 3% | .04 |

(a) N = no size effect; S = small size effect; M = medium size effect; L = large size effect
