# Supplementary material for: A Digital Program for Daily Life Management With Endometriosis: Pilot Cohort Study on Symptoms and Quality of Life Among Participants
Source: JMIR Form Res. 2025 Feb 28;9:e58262. doi: 10.2196/58262 (PMC11909486; doi:10.2196/58262)
Supplement: Multimedia Appendix 3 [file formative_v9i1e58262_app3.docx]

Multimedia appendix 3. Evolution of outcomes between baseline and 3 months for all endometriosis program participants according to the sections thresholds of the program tested.

|  | **QoL - EHP-5 - core** | | | | | **Global symptom burden** | | | | |
| --- | --- | --- | --- | --- | --- | --- | --- | --- | --- | --- |
|  | **N** | **Improvement (%)** | **Stable (%)** | **Deterioration (%)** | ***P*** | **N** | **Improvement (%)** | **Stable (%)** | **Deterioration (%)** | ***P*** |
| **Number of sections consulted** |  | | | | |  | | | | |
| Not all sections consulted | 20 | 0% | 85% | 15% | <.001 | 52 | 8% | 92% | 0% | 0.02 |
| All sections consulted | 36 | 47% | 36% | 17% |  | 82 | 20% | 74% | 6% |  |
| **Number of action-oriented contents consulted** |  | | | | |  | | | | |
| Action-oriented contents not consulted | 21 | 14% | 67% | 19% |  | 54 | 9% | 87% | 4% |  |
| Action-oriented contents consulted in part | 14 | 36% | 57% | 7% | 0.22 | 43 | 16% | 79% | 5% | 0.53 |
| All action-oriented contents consulted | 21 | 43% | 38% | 19% |  | 37 | 21% | 76% | 3% |  |
