# Supplementary material for: A Digital Program for Daily Life Management With Endometriosis: Pilot Cohort Study on Symptoms and Quality of Life Among Participants
Source: JMIR Form Res. 2025 Feb 28;9:e58262. doi: 10.2196/58262 (PMC11909486; doi:10.2196/58262)
Supplement: Multimedia Appendix 4 [file formative_v9i1e58262_app4.docx]

**Multimedia Appendix 4.** **Beta coefficients and 95% confidence intervals of non-adjusted linear regression models evaluating associations between endometriosis program participation and health outcomes, and interaction tests according to baseline quality of life levels (EHP-5 core).**

| **Reference**  program participant | **β (95% CI)** | **P_interaction_** | **Low QoL**  **β (95% CI)**  (n=21 participant, n=25 control) | **Medium QoL**  **β (95% CI)**  (n=41 participant, n=66 control) | **Good QoL**  **β (95% CI)**  (n=30 participant, n=58 control) |
| --- | --- | --- | --- | --- | --- |
|  |  |  |  |  |  |
| **Global symptom burden** | **0.40 (0.03 ; 0.77)** | 0.2251 |  |  |  |
| **Overall pain** | -0.01 (-0.70 ; 0.69) | 0.5030 |  |  |  |
| **Anxiety** | **1.25 (0.56 ; 1.93)** | 0.9894 |  |  |  |
| **Depression** | **0.88 (0.13 ; 1.63)** | 0.0676 |  |  |  |
| **Dysmenorrhea** | **1.23 (0.20 ; 2.25)** | 0.7343 |  |  |  |
| **Dyspareunia** | -0.19 (-1.09 ; 0.71) | 0.5191 |  |  |  |
| **Dyschezia** | -0.20 (-0.88 ; 0.48) | **0.0360** | -0.21 (-1.66 ; 1.24) | 1.05 (-0.13 ; 2.23) | **-1.73 (-2.60 ; -0.85)** |
| **Dysuria** | 0.34 (-0.26 ; 0.94) | 0.9662 |  |  |  |
| **Chronic pelvic pain** | 0.24 (-0.48 ; 0.97) | 0.5197 |  |  |  |
| **Gastrointestinal disorders** | 0.06 (-0.58 ; 0.70) | 0.4174 |  |  |  |
| **Chronic fatigue** | 0.49 (-0.14 ; 1.12) | 0.3242 |  |  |  |
| **Neuropathic pain** | **0.96 (0.26 ; 1.65)** | 0.7126 |  |  |  |
| **Endobelly** | 0.51 (-0.16 ; 1.18) | 0.6170 |  |  |  |
| **QoL - EHP-5 - core** | 6.90 (-0.08 ; 13.88) | **0.0018** | **12.00 (1.36 ; 22.64)** | **11.38 (3.77 ; 18.99)** | **-22.58 (-41.53 ; -3.64)** |
| **QoL - EHP-5 - modular** | **5.69 (0.23 ; 11.14)** | 0.1568 |  |  |  |
| **QoL - EQ-5D score** | **-0.08 (-0.01 ; -0.03)** | 0.4648 |  |  |  |

If β < 0, the outcome shows greater improvement in program participants compared to controls (except for EQ-5D, where the interpretation is reversed).
